# Nitric oxide acts upstream of ethylene in cell wall phosphorus reutilization in phosphorus-deficient rice

**DOI:** 10.1093/jxb/erw480

**Published:** 2017-01-07

**Authors:** Xiao Fang Zhu, Chun Quan Zhu, Chao Wang, Xiao Ying Dong, Ren Fang Shen

**Affiliations:** State Key Laboratory of Soil and Sustainable Agriculture, Institute of Soil Science, Chinese Academy of Science, Nanjing, China

**Keywords:** Cell wall, ethylene, NO, *Oryza sativa*, phosphorus, pectin, remobilization, translocation

## Abstract

Nitric oxide (NO) and ethylene are both involved in cell wall phosphorus (P) reutilization in P-deficient rice; however, the crosstalk between them remains unclear. In the present study using P-deficient ‘Nipponbare’ (Nip), root NO accumulation significantly increased after 1 h and reached a maximum at 3 h, while ethylene production significantly increased after 3 h and reached a maximum at 6 h, indicating NO responded more quickly than ethylene. Irrespective of P status, addition of the NO donor sodium nitroprusside (SNP) significantly increased while the NO scavenger 2-(4-carboxyphenyl)-4,4,5,5-tetramethylimidazoline-1-oxyl-3-oxide (c-PTIO) significantly decreased the production of ethylene, while neither the ethylene precursor 1-aminocyclopropane-1-carboxylic acid (ACC) nor the ethylene inhibitor aminoethoxyvinylglycine (AVG) had any influence on NO accumulation, suggesting NO acted upstream of ethylene. Under P-deficient conditions, SNP and ACC alone significantly increased root soluble P content through increasing pectin content, and c-PTIO addition to the ACC treatment still showed the same tendency; however, AVG+SNP treatment had no effect, further indicating that ethylene was the downstream signal affecting pectin content. The expression of the phosphate transporter gene *OsPT2* showed the same tendency as the NO–ethylene–pectin pathway. Taken together, we conclude that ethylene functions downstream of NO in cell wall P reutilization in P-deficient rice.

## Teaser

Ethylene functions downstream of nitric oxide in cell wall phosphorus reutilization in P-deficient rice through controlling pectin biosynthesis and expression of phosphate transporter gene *OsPT2.*

## Introduction

As one of the major plant macronutrients, phosphorus (P) is not only essential for the structure of cell components such as DNA, RNA, ATP and membranes, but also involved in plant development and metabolism (Marschner, 1995). Despite high P in the soil, inorganic phosphorous (P_i_) is one of the least available nutrients for crops as much of it is converted to organic matter by microorganisms or is bound to cations, and thus difficult for plants to absorb (Tiessen, 2008; Wu *et al.*, 2013). In fact, the concentration of P_i_ available in soil rarely exceeds 10 μM (Shen *et al.*, 2011), which is barely sufficient to meet plants’ needs. Therefore, it is desirable to investigate the mechanisms by which plants can survive under conditions of P starvation, to develop new crops that can adapt to low P conditions.

One of the most common strategies used by plants to cope with low P_i_ availability is to enhance P_i_ acquisition from soil by remodeling the architecture and morphology of the root system. This can be done, for example, by growing more lateral roots in shallow soils, inhibiting elongation of the primary root, increasing the number of root hairs and root branches (López-Bucio *et al.*, 2003; Desnos, 2008), forming symbiotic associations with mycorrhizal fungi (Brundrett, 2002; Boulet and Lambers, 2005), or forming ‘root clusters’ (Shane and Lambers, 2005; Lynch and Brown, 2008; Vance, 2008). Plants may also secrete root exudates, such as carboxylates (Keerthisinghe *et al.*, 1998), phenolics (Juszczuk *et al.*, 2004), mucilage (Grimal *et al.*, 2001), and phosphatases (Wasaki *et al.*, 2003; Abram *et al.*, 2009) to change the chemical and biological properties of the soil, thus facilitating the release of insoluble P from the soil and allowing it to be utilized by the plant. Another strategy is to relocate and reutilize internal P_i_, for instance, by altering metabolic pathways, remodeling lipids, or enhancing ribonuclease (RNase) activity (Liang *et al.*, 2014). Zhu *et al.* (2015) described a pectin-regulated strategy in rice in which the pectin content is increased to compete with iron (Fe) from FePO_4_ and root cell wall P is reutilized under P-limited conditions.

As a stress phytohormone, ethylene is involved in plant responses to various biotic or abiotic stresses, such as heat stress (Clarke *et al.*, 2009), ozone stress (Vahala *et al.*, 2003) and nutrient toxicity or deficiency (Iqbal *et al.*, 2013). Li *et al.* (2009) reported a significant increment of ethylene emission under P-deficient conditions, which in turn modified the root hydraulic conductivity. Furthermore, addition of 1-aminocyclopropane-1-carboxylic acid (ACC) to the roots of P-sufficient plants leads to the same root architecture observed in plants under P-deficient conditions, e.g. increased root hair formation (Tanimoto *et al.*, 1995). Ethylene is also involved in cell wall P remobilization in rice to maintain internal P_i_ homeostasis (Zhu *et al.*, 2016*a*); however, it is unknown whether other signaling molecules regulate ethylene in this root cell wall P reutilization pathway.

NO is a signaling molecule that is involved in diverse physiological processes throughout the plant life cycle. NO functions in responses to abiotic stresses (Qiao *et al.*, 2014), including salt stress in rice (Uchida *et al.*, 2002), ultraviolet radiation in maize (Wang *et al.*, 2006) and heavy metal stress in wheat (Singh *et al.*, 2008). In white lupin (*Lupinus albus*), under P-deficient conditions, root NO accumulation is significantly increased, which in turn stimulates the exudation of citrate to enhance external P acquisition, thus leading to improved growth (Wang *et al.*, 2010; Meng *et al.*, 2012). In our previous study, we found that NO is involved in rice root cell wall P remobilization (Zhu *et al.*, 2016*b*), and now, in the present study, we used rice cultivar ‘Nipponbare’ (Nip) to study whether there is crosstalk between NO and ethylene in internal cell wall P remobilization under P-deficient conditions. This study provides the first description of an upstream regulatory mechanism of ethylene for cell wall P reutilization under P-deficient conditions in rice.

## Materials and methods

### Plant material and cultivation conditions

Seeds of rice (*Oryza sativa*) spp. *japonica* ‘Nipponbare’ (‘Nip’) were first dipped in 1% NaClO, then incubated in water after thoroughly washing with deionized water. Two days later, seeds were cultivated on a plastic supporting net (about 2 mm^2^) in a plastic container containing a 0.5 mM CaCl_2_ (pH 5.5) solution. After another 2 d, this solution was then replaced with full strength Kimura B solution in accordance with Zhu *et al* (2016*b*). Seedlings were grown under controlled conditions: photosynthetically active radiation (PAR) of 400 µmol m^–2^ s^–1^ light intensity, 60% relative humidity, day/night cycles of 14 h days at 26 °C and 10 h nights at 23 °C. The vertical height of the uppermost leaves was about 20 cm when the PAR measurement was undertaken.

To measure NO content and the production of ethylene, 2-week-old seedlings were grown under P-deficient (–P) conditions. Rice roots were collected at 0 (+P control), 1, 3, 6, and 12 h and 1, 2, 3, 4, 5, 6, and 7 d. To investigate which hormone acts upstream of cell wall P reutilization, 2-week-old seedlings were transferred to the following treatments: +P, +P + sodium nitroprusside (SNP), +P + 2-(4-carboxyphenyl)-4,4,5,5-tetramethylimidazoline-1-oxyl-3-oxide (c-PTIO), –P, –P + SNP, and –P + c-PTIO. Since the production of ethylene reached its maximum value at 6 h after –P treatment, rice roots were collected after 6 h of treatment, and ethylene production was measured. A further six groups of rice plants were transferred to 1.5-liter pots with the following treatments: +P, +P + 1-aminocyclopropane-1-carboxylic acid (ACC), +P + aminoethoxyvinylglycine (AVG), –P, –P + ACC, and –P + AVG. Rice roots were collected and NO content was measured 3 h after treatment because NO content reached its maximum value after 3 h of –P treatment. To investigate soluble P content and to extract cell walls, 2-week-old seedlings were transferred to the following 10 treatments: +P, +P + ACC, +P + SNP, +P + ACC + c-PTIO, +P + SNP + AVG, –P, –P + ACC, –P + SNP, –P + ACC + c-PTIO, and –P + SNP + AVG. The final concentrations were 1 μM ACC, 2.5 μM SNP, 10 μM c-PTIO, and 0.2 μM AVG. As SNP was applied as pretreatment, the nutrient solution was renewed after 24 h with P-deficient or P-sufficient solutions containing other substances for another 6 d. The pH was adjusted to 5.6 and the corresponding treated nutrient solution was renewed every 3 d.

### Measurement of soluble P_i_ content

After washing three times with deionized water, roots and shoots were separated and weighed immediately, then ground with 5 M sulfuric acid. After a 10-fold dilution with deionized water, material was centrifuged at 12 000 *g* for 10 min, and 400 μl supernatant was mixed with 200 μl ammonium molybdate containing 15% fresh ascorbic acid (pH 5.0) for 30 min. Absorption values were determined at 650 nm, and the final P_i_ concentration was calculated per gram fresh weight (Zheng *et al.*, 2009).

### Extraction and fractionation of cell walls

A previously described method of Zhong and Lauchli (1993) was used for cell wall extraction. First, about 0.05 g fresh weight of roots was ground in liquid nitrogen, and then washed with 8 ml 75% ethanol, 8 ml acetone, 8 ml 1:1 methanol:chloroform and 8 ml methanol, respectively, and incubated for 20 min. The final solution was centrifuged at 3256 *g* for 10 min at 4 °C, and the pellets were dried and stored at 4 °C for further use.

Extraction of pectin was carried out as follows: about 2 mg cell walls was weighed into a 1.5 ml tube, and 1 ml deionized water was added. Then this suspended solution was incubated in a 100 °C water bath for 1 h. After centrifuged at 12 000 g for 10 min, supernatants were collected in a 5-ml tube. This procedure was repeated three times (Zhong and Lauchli, 1993).

### Measurement of pectin content and pectin methylesterase activity

Pectin content was estimated by the concentration of uronic acid. Briefly, 200 μl pectin solution was incubated with 1 ml 98% H_2_SO_4_ containing 12.5 mM Na_2_B_4_O_7_·10H_2_O at 100 ºC in a water bath for 5 min. After cooling, 20 μl of 0.15% *m*-hydroxydiphenyl was added and absorbance was measured at 520 nm with galacturonic acid as the standard.

For measurement of pectin methylesterase (PME) activity, about 5 mg cell walls was first suspended in 1 M NaCl solution (pH 6.0) at 4 °C for 1 h with repeated vortexing (20 s for 10 min each). Then the extracts were centrifuged at 16 800 *g* for 10 min to collect the supernatant. Finally, after 50 μl of supernatant was incubated with 10 μl alcohol oxidase and 100 μl 200 mM phosphate buffer (0.2 M Na_2_HPO_4_:0.2 M NaH_2_PO_4_ , 21:4, v/v) containing 0.64 mg ml^–1^ pectin at 30 °C for 10 min, 200 μl 0.5 M NaOH containing 5 mg ml^–1^ Purpald was added and absorbance was measured at 550 nm with methanol as the standard.

### Measurement of cell wall P content

Cell wall P concentration was determined using the following steps: approximately 2 mg cell walls was shaken with 1 ml 2 M HCl in a 1.5 ml tube. After 24 h, a sample was centrifuged, and the supernatant was collected for P concentration determination (Zhu *et al.*, 2015).

### Measurement of root NO content

Accumulation of endogenous NO in rice roots was assayed using 10 μM 4-amino-5-methylamino-2,7-diﬂuoroﬂuorescein diacetate (DAF-FM DA). The apical 1 cm of root tips was collected and washing with HEPES–KOH (pH 7.4). After 15 min, root tips were incubated with 500 μl DAF-FM DA in darkness for 30 min. Then, fresh buffer was used to wash root tips three times to remove excess ﬂuorescent dye. A Nikon Eclipse 80i light microscope was used to visualize the NO fluorescence. The intensity of fluorescence was measured using Adobe Photoshop 7.0 software (Besson-Bard *et al.*, 2009).

### Measurement of ethylene emission

Emission of ethylene from rice roots was analysed according to Wu *et al.* (2011). Briefly, rice roots were detached and transferred to 15 ml glass vials that contained 1 ml distilled water, and the vials were immediately sealed with a rubber stopper. After 2 h incubation in darkness at 30 °C, 1 ml gas from each vial was measured according to Zhu *et al* (2016*a*).

### Analysis of relative gene expression

After treatment for 1 week, roots were collected and immediately transferred to liquid nitrogen. RNA was extracted and reverse-transcribed according to Zhu *et al* (2016*b*). Agarose gel electrophoresis and spectroscopy were used to guarantee the integrity and quality of RNA. The total volume of the real-time PCR mixture was 10 µl, made up of 5 µl SYBR Premix ExTaq, 0.6 µl forward primers, 0.6 µl reverse primers, 2.8 µl sterile distilled water and 1 µl cDNA (10-fold dilution). Each cDNA sample was run in triplicate. Primers used in the present study are given in Supplementary Table S1 at *JXB* online (Ai *et al.*, 2009; Jia *et al.*, 2011). *OsHistone H3* (Huang *et al.*, 2012) was used to confirm that the expression of the reference gene *OsACTIN* was independent of all treatments (see Supplementary Fig. S1).

### Statistical analysis

All experiments were conducted at least in triplicate. One-way ANOVA was used to analyse the data, and the mean values were compared using Duncan’s multiple range test. Letters on the figures presented here indicate that the mean values were statistically different at *P*<0.05.

## Results

To verify the effects of NO and ethylene on rice, 2-week-old Nip seedlings were transferred to +P or –P nutrient solutions. As shown in Supplementary Fig. S2, more soluble P was found in roots and shoots when seedlings were pretreated with SNP or when ACC was applied to the –P nutrient solution, implying that both NO and ethylene are involved in internal P reutilization in Nip. In our previous study, we found that approximately 50% of the total P in Nip roots was deposited in cell walls and pectin played pivotal roles in root cell wall P reutilization (Zhu *et al.*, 2015). As expected, in the present study, less P was retained in the root cell walls, and cell wall pectin content increased when plants were pretreated with SNP or when ACC was applied to the nutrient solution under –P conditions (see Supplementary Fig. S3). This finding corroborates our previous studies (Zhu *et al.*, 2016a, b), indicating that both NO and ethylene are involved in the reutilization of cell wall-deposited P through regulating pectin content.

**Fig. 1.**
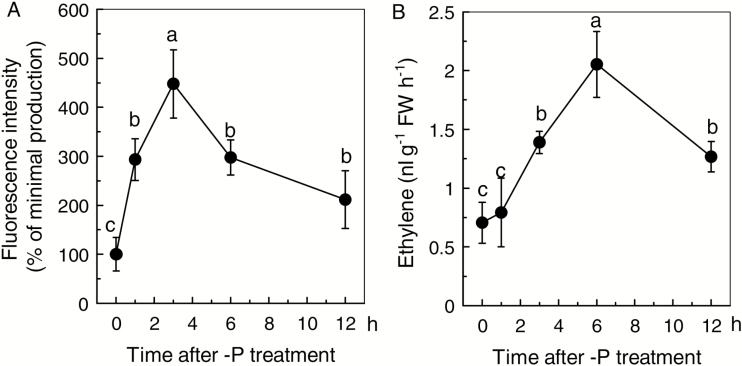
Effect of P deficiency on NO production (A) and ethylene emission (B) in rice root. NO production is indicated by green fluorescence and expressed as relative fluorescence intensity (% of minimal production). Data are means+SD (*n*=10). Columns with different letters show significant differences at *P*<0.05. FW: fresh weight.

**Fig. 2.**
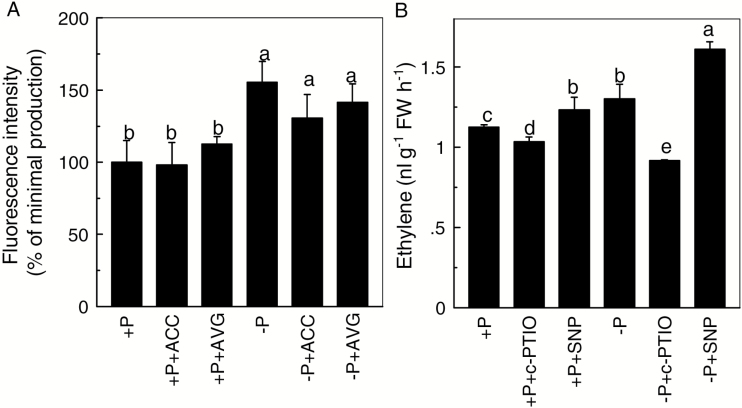
Effect of different treatments on NO production (A) and ethylene emission (B) in rice root. NO production is indicated by green fluorescence and expressed as relative fluorescence intensity (% of minimal production). Data are means+SD (*n*=10). Columns with different letters show significant differences at *P*<0.05.

**Fig. 3.**
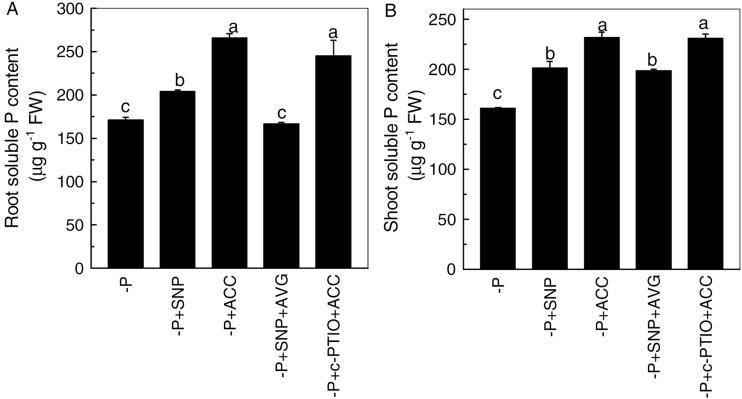
Effect of different treatments on root (A) and shoot (B) soluble P content in P-deficient rice. Data are means+SD (*n*=4). Columns with different letters show significant differences at *P*<0.05.

As both NO and ethylene act upstream of cell wall pectin, the question was raised whether ethylene acts through NO, NO acts through ethylene, or both act in conjunction. To resolve this, 2-week-old normally cultivated (+P) Nip seedlings were transferred to a completely P-deficient solution, and the production of NO and ethylene was measured at 0 (+P as control), 1, 3, 6, and 12 h. As shown in Fig. 1A and Supplementary Fig. S4, NO content significantly increased after 1 h –P treatment and reached its maximum value at 3 h. However, the production of ethylene increased after 3 h –P treatment and reached its maximum value at 6 h (Fig. 1B). This conclusion was further confirmed by the measurement of NO and ethylene content for the remainder of the experimental period. Compared with the 12 h –P treatment, both NO and ethylene content were similar or even lower than the 7 d –P treatment (see Supplementary Fig. S5). These findings suggest that NO responded more quickly than ethylene when plants were starved of P. This finding in turn raised the question of whether the increased accumulation of NO acts as the signal to increase the production of ethylene. As shown in Fig. 2B, irrespective of P status, the NO donor SNP significantly increased while the NO scavenger c-PTIO significantly decreased the production of ethylene, suggesting that NO may act upstream of ethylene. This hypothesis was further confirmed by the finding that the ethylene precursor ACC and inhibitor AVG had no effect on NO accumulation in rice roots, neither under P-sufficient nor under P-deficient conditions (Fig. 2A and Supplementary Fig. S6). Moreover, in the presence of ACC, root and shoot soluble P content was unaffected by c-PTIO when compared with the –P + ACC treatment (Fig. 3). However, when compared with the –P + SNP treatment, the root soluble P content was decreased when AVG was applied in combination with SNP (Fig. 3A), indicating that decreased ethylene production by exogenous application of AVG counteracts and overrides the synergistic effect resulting from the increment of NO content. These results suggest that ethylene acts downstream of NO in this internal P reutilization pathway.

**Fig. 4.**
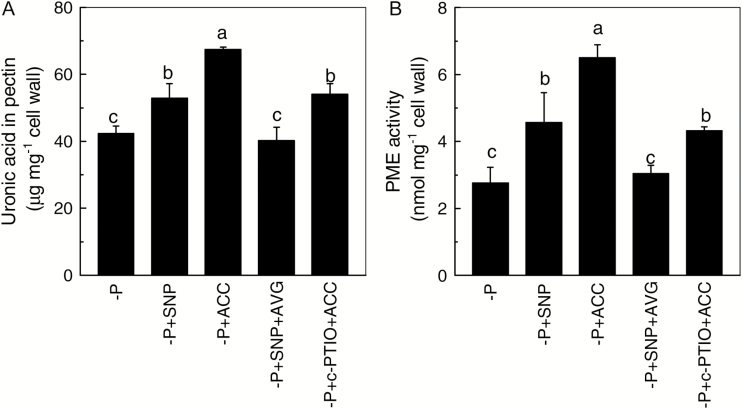
Effect of different treatments on cell wall pectin content (A) and pectin methylesterase (PME) activity (B) in P-deficient rice. Data are means+SD (*n*=4). Columns with different letters show significant differences at *P*<0.05.

As cell wall pectin contributes greatly to the internal P reutilization in P-deficient rice (Zhu *et al.*, 2015), we aimed to further clarify the relationship between NO, ethylene and cell wall pectin by combining AVG and SNP treatments. We found that the inhibition of ethylene production (caused by application of AVG) abolished the effect of SNP treatment alone and thus decreased the pectin content to a level as low as –P treatment alone. However, c-PTIO application did not reverse the increment of pectin content in the presence of ACC (Fig. 4A). This result suggests that ethylene acts downstream of NO to regulate the root cell wall pectin content in P-deficient rice. Only de-esterified pectin will present a net negative charge in the cell wall space, and this negative charge is generated from the demethylation of the pectin, which is catalysed by methylesterase (PME). Accordingly, we measured PME activity and found that it showed the same tendency as the pectin content (Fig. 4B), indicating that the pectin that accumulates in response to NO signaling mediated by ethylene is enriched in de-esterified pectin.

**Fig. 5.**
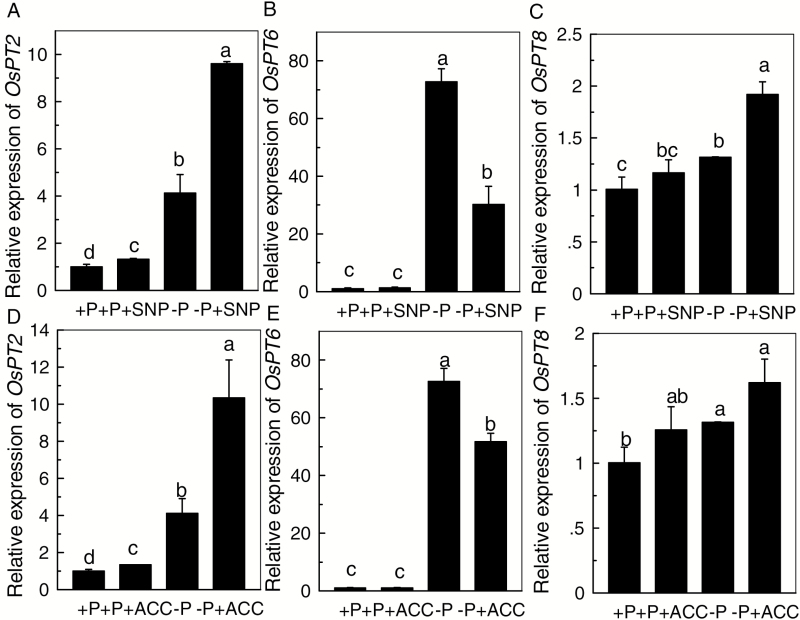
Effect of sodium nitroprusside (SNP) on the expression of *OsPT2* (A), *OsPT6* (B), and *OsPT8* (C), and effect of 1-aminocyclopropane-1-carboxylic acid (ACC) on the expression of *OsPT2* (D), *OsPT6* (E), and *OsPT8* (F) in rice roots under P-sufficient (+P) or P-deficient (–P) conditions. *OsACTIN* was used as the reference gene. Data are means+SD (*n*=4). Columns with different letters show significant differences at *P*<0.05.

**Fig. 6.**
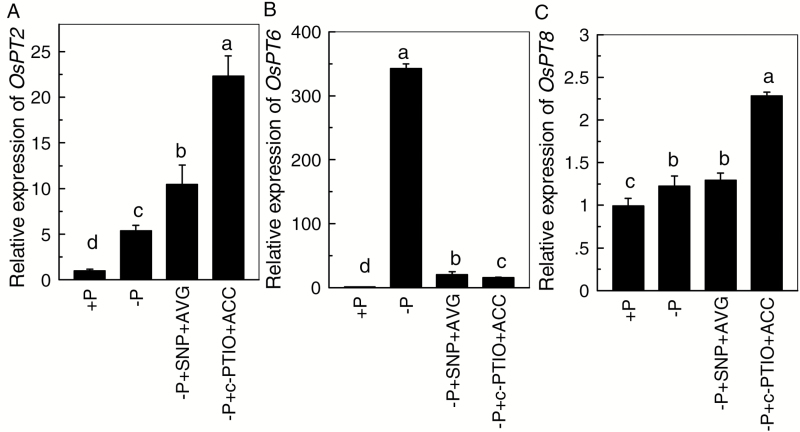
Effect of different treatments on the expression of *OsPT2* (A), *OsPT6* (B), and *OsPT8* (C) in P-deficient rice. *OsACTIN* was used as the reference gene. Data are means+SD (*n*=4). Columns with different letters show significant differences at *P*<0.05.

To determine if NO and ethylene might affect the translocation of P in rice under P-starvation conditions, three candidate genes (*OsPT2*, *OsPT6*, and *OsPT8*) responsible for P translocation were analysed and *OsACTIN* was used as the reference gene. When Nip was cultivated in P-sufficient conditions, the addition of SNP or ACC alone significantly increased the expression of *OsPT2* in root cells, but had almost no influence on the expression of *OsPT6* or *OsPT8* (Fig. 5). This was also true when Nip was cultivated in –P conditions. These findings indicate that *OsPT2* may be associated with the role of NO or ethylene in alleviating P deficiency (Fig. 5). Moreover, as shown in Fig. 6, compared with the SNP + AVG treatment, c-PTIO combined with ACC treatment resulted in higher expression of *OsPT2*. This pattern held true when *OsHistone H3* was used as the reference gene (see Supplementary Figs S7 and S8), further indicating that ethylene, rather than NO, is the more downstream signal that induced the expression of *OsPT2* in P-deficient rice.

## Discussion

As a signaling molecule and a stress phytohormone, NO and ethylene are involved in a variety of stress responses (Morgan and Drew, 1997; Delledonne *et al.*, 1998), such as Fe deficiency (Wu *et al.*, 2011; Zhu *et al.*, 2016*c*). In our previous study, we found that both ethylene and NO were involved in the P-deficiency response in rice (Zhu *et al.*, 2016a, b). In the present study, after pretreatment with SNP, or when ACC was applied, rice root and shoot soluble P contents were significantly increased, irrespective of P status (see Supplementary Fig. S2). This finding indicates that NO and ethylene do indeed take part in the internal reutilization of P in rice, especially under –P conditions. Since the effect of NO on internal P reutilization parallels that of ethylene, it is possible that there is crosstalk between them. Accumulating evidence indicates that ethylene acts downstream of NO and that ethylene production can be stimulated by NO in response to abiotic stresses such as ozone stress in tobacco (Ederli *et al.*, 2006) and ultraviolet B stress in maize seedlings (Wang *et al.*, 2006). In the present study, when Nip was grown in the –P nutrient solution, NO accumulated more quickly than ethylene in the root (Fig. 1 and Supplementary Fig. S4), and the emission of ethylene was significantly increased under SNP pretreatment and notably decreased under c-PTIO treatment (Fig. 2B). However, ACC and AVG failed to induce the accumulation of NO (Fig. 2A and Supplementary Fig. S6), indicating that NO acts as an upstream signal to induce the emission of ethylene in rice under –P stress. This conclusion was strengthened by the fact that treatment with SNP and AVG failed to increase root soluble P, but treatment with ACC and c-PTIO increased root and shoot soluble P (Fig. 3), indicating that ethylene acts downstream of NO.

Where does the internal P come from in the P-deficient rice? As demonstrated in our previous study, about 50% of the rice root total P is stored in the cell wall (Zhu *et al.*, 2016*a*). Thus we hypothesized that the incremental increase in soluble P by SNP or ACC treatment may result from the remobilization of the cell wall P. This hypothesis was confirmed (see Supplementary Fig. S3B, D. Although cellulose, hemicellulose and pectin are the main components of plant cell walls, only pectin has been shown to be involved in plant response to P starvation. For example, compared with other plants, groundnut possesses a superior ability to acquire soil P in P-deficient soil because its root cell wall has ‘contact reaction’ pectin (Ae and Shen, 2002). Recently, Zhu *et al* (2015) reported that pectin is able to reutilize cell wall P by using its negative charges (–COO^–^) to bind cations such as Al or Fe, thus facilitating the release of cell wall P. It is interesting that the content of pectin in the rice root cell wall can be regulated by signaling molecules such as NO under Cd toxicity (Xiong *et al.*, 2009) and by ethylene under P-deficient conditions (Zhu *et al.*, 2016*a*). In the present study, under –P conditions, when SNP or ACC was applied exogenously, an incremental change in cell wall pectin content was observed in association with a notable reduction in cell wall P content (see Supplementary Fig. S3). This indicates that both NO and ethylene take part in the reutilization of cell wall P to maintain internal P homeostasis, and provides an opportunity to further investigate which hormone acts upstream of the other. A combination of SNP and AVG treatment decreased the de-esterified cell wall pectin content to a level as low as under –P treatment alone, while combined c-PTIO and ACC treatment still increased de-esterified pectin content (Fig. 4). This finding further indicates that the regulation of pectin content is largely dependent on ethylene, while NO acts upstream of ethylene in the P-deficient rice.

Regulation of the translocation of internal soluble P is also very important for resistance of rice to P deficiency. Three rice P_i_:H^+^ cotransporters (PHTs) have been shown to be essential for the translocation of P from the root to the shoot (Ai *et al.*, 2009; Jia *et al.*, 2011). In the present study, under –P conditions, SNP and ACC markedly enhanced the expression of *OsPT2* (Fig. 5), implying that NO and ethylene signaling may share the same pathway. Furthermore, higher expression of *OsPT2* was observed in the –P + c-PTIO + ACC treatment when compared with –P + SNP + AVG (Fig. 6), indicating that ethylene is the more downstream signal in the regulation of the expression of *OsPT2* relative to NO. The regulatory behavior of ethylene is mainly attributed to 12 ethylene-responsive element-binding factors (with a GCCGCC motif) located in the *OsPT2* promoter (Chakravarthy *et al.*, 2003). Furthermore, compared with –P treatment alone, the increased shoot soluble P content observed under –P + SNP + AVG treatment may be attributed to the significantly increased expression of *OsPT2* and *OsPT8* (Figs 3B and 6).

**Fig. 7.**
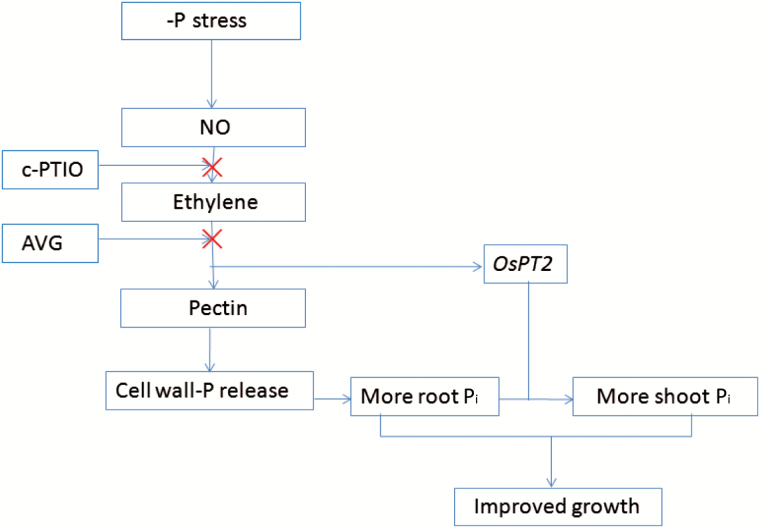
Model illustrating the hypothetical function of ethylene in NO-mediated root cell wall P reutilization in rice (*Oryza sativa*) under –P conditions. Red cross indicates inhibition of the pathway.

Based on current results and our previous work, we propose a model shown in Fig. 7. When rice suffers P deficiency, NO responds quickly and induces the production of ethylene. Then, on the one hand, pectin content is increased to reutilize cell wall P, and on the other hand, the expression of *OsPT2* is up-regulated to facilitate the translocation of P from root to shoot. Thus, the growth of rice under P-deficient conditions is improved.

## Supplementary data

Supplementary data are available at *JXB* online. 

Fig. S1. Effect of sodium nitroprusside (SNP), 1-aminocyclopropane-1-carboxylic acid (ACC), and different treatments on the expression of the reference gene *OsACTIN* in rice roots under +P or –P condition.

Fig. S2. Effect of SNP on root soluble P content, shoot soluble P content, and effect of ACC on root soluble P content and shoot soluble P content under +P or –P condition.

Fig. S3. Effect of SNP on cell wall pectin content, cell wall P content and effect of ACC on cell wall pectin content, cell wall P content in rice root under +P or –P condition.

Fig. S4. Effect of P deficiency on NO production in rice root.

Fig. S5. Effect of P deficiency on NO production and ethylene emission in rice root.

Fig. S6. Effect of different treatments on NO production in rice root under P-sufficient and P-deficient condition.

Fig. S7. Effect of SNP on the expression of *OsPT2*, *OsPT6*, and *OsPT8*, and effect ACC on the expression of *OsPT2*, *OsPT6*, and *OsPT8* in rice roots under +P or –P condition.

Fig. S8. Effect of different treatments on the expression of *OsPT2*, *OsPT6*, and *OsPT8* in P-deficient rice.

Table S1. Gene-specific primers used in this work.

## Supplementary Material

supplementary_table_S1_figures_S1_S8
