# Supplementary material for: Nitric oxide acts upstream of ethylene in cell wall phosphorus reutilization in phosphorus-deficient rice
Source: J Exp Bot. 2017 Jan 7;68(3):753–60. doi: 10.1093/jxb/erw480 (PMC6055659; doi:10.1093/jxb/erw480)
Supplement: supplementary_table_S1_figures_S1_S8 [file erw480_suppl_supplementary_table_s1_figures_s1_s8.pdf]

Title: Nitric oxide acts upstream of ethylene in cell wall phosphorus reutilization in phosphorus-deficient rice (*Oryza sativa*)

Authors: Xiao Fang Zhu, Chun Quan Zhu, Chao Wang, Xiao Ying Dong and Ren Fang Shen*

State Key Laboratory of Soil and Sustainable Agriculture, Institute of Soil Science, Chinese Academy of Science, Nanjing 210008, China

***Corresponding author：**

Name: Ren Fang Shen

Email: rfshen@issas.ac.cn

Running Title： Nitric oxide acts upstream of ethylene in P-deficient rice

Supplemental Table 1 Gene-specific primers used in this work.

Gene	Forward (5'-3')	Reverse (5'-3')
OsPT2	GACGAGACCGCCCAAGAAG	TTTTCAGTCACTCACGTCGAGAC
OsPT6	TATAACTGATCGATCGAGACCAGAG	TGGATAGCCAGGCCAGTTATATATC
OsPT8	AGAAGGCAAAAGAAATGTGTGTTAAAT	AAAATGTATTCGTGCCAAATTGCT
OsACTIN	TCCGGTGGATCTTCATGCTTACCT	ATGGACCATTGCGACGAGTCTTCT
OsHistone H3	GGTCAACTTGTTGATTCCCCTCT	AACCGCAAAATCCAAAGAACG

Supplemental Figure Legends

Supplemental Figure 1 Effect of sodium nitroprusside (SNP) (A), 1-aminocyclopropane-1-carboxylic acid (ACC) (B) and different treatments (C) on the expression of the reference gene-*OsACTIN* in rice roots under +P or -P condition. *OsHistone H3* was used as the reference gene. Data are means ± SD (n = 4). Columns with different letters show significant differences at P < 0.05.

Supplemental Figure 2 Effect of SNP on root soluble P content (A), shoot soluble P content (B), and effect of ACC on root soluble P content (C) and shoot soluble P content (D) under +P or -P condition. Data are means ± SD (n = 4). Columns with different letters show significant differences at P < 0.05. FW: fresh weight.

Supplemental Figure 3 Effect of SNP on cell wall pectin content (A), cell wall P content (B) and effect of ACC on cell wall pectin content (C), cell wall P content (D) in rice root under +P or -P condition. Data are means ± SD (n = 4). Columns with different letters show significant differences at P < 0.05.

Supplemental Figure 4 Effect of P deficiency on NO production in rice root. Seedlings were first grown in the P sufficient condition as control (A), and then subjected to P deficient condition for 1 h (B), 3 h (C), 6 h (D) and 12 h (E). Root tips were collected for the NO fluorescence measurement. Data are means ± SD (n = 10). Scale bar = 1 mm.

Supplemental Figure 5 Effect of P deficiency on NO production (A) and ethylene emission (B) in rice root. NO production is indicated by green fluorescence and expressed as relative fluorescence intensity (% of minimal production). Data are means ± SD (n = 10). Columns with different letters show significant differences at P < 0.05. FW: fresh weight.

Supplemental Figure 6 Effect of different treatments on NO production in rice root under P sufficient (A, B and C) and P deficient condition (D, E and F). (B) and (D) seedlings were treated with 1 μM ACC, (C) and (F) seedlings were treated with 0.2 μM aminoethoxyvinylglycine (AVG). The root tips were collected for the NO

fluorescence measurement. Data are means ± SD (n = 10). Scale bar = 1 mm.

Supplemental Figure 7 Effect of SNP on the expression of *OsPT2* (A), *OsPT6* (B) and *OsPT8* (C), and effect ACC on the expression of *OsPT2* (D), *OsPT6* (E) and *OsPT8* (F) in rice roots under +P or -P condition. *OsHistone H3* was used as a reference gene. Data are means ± SD (n = 4). Columns with different letters show significant differences at $P < 0.05$.

Supplemental Figure 8 Effect of different treatments on the expression of *OsPT2* (A), *OsPT6* (B) and *OsPT8* (C) in P-deficient rice. *OsHistone H3* was used as the reference gene. Data are means ± SD (n = 4). Columns with different letters show significant differences at $P < 0.05$.

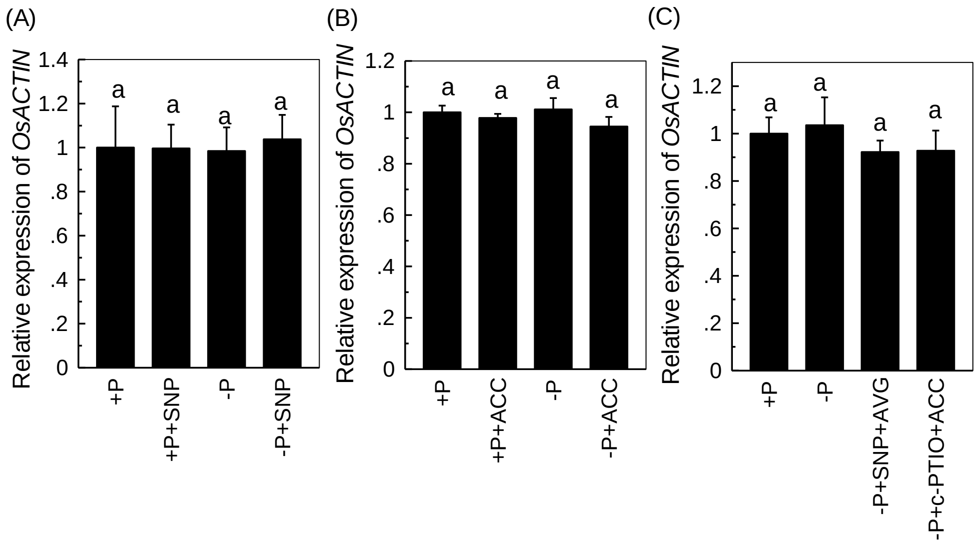

Supplemental Figure 1 Effect of sodium nitroprusside (SNP) (A), 1-aminocyclopropane-1-carboxylic acid (ACC) (B) and different treatments (C) on the expression of the reference gene-*OsACTIN* in rice roots under +P or -P condition. *OsHistone H3* was used as the reference gene. Data are means ± SD (n = 4). Columns with different letters show significant differences at $P < 0.05$.

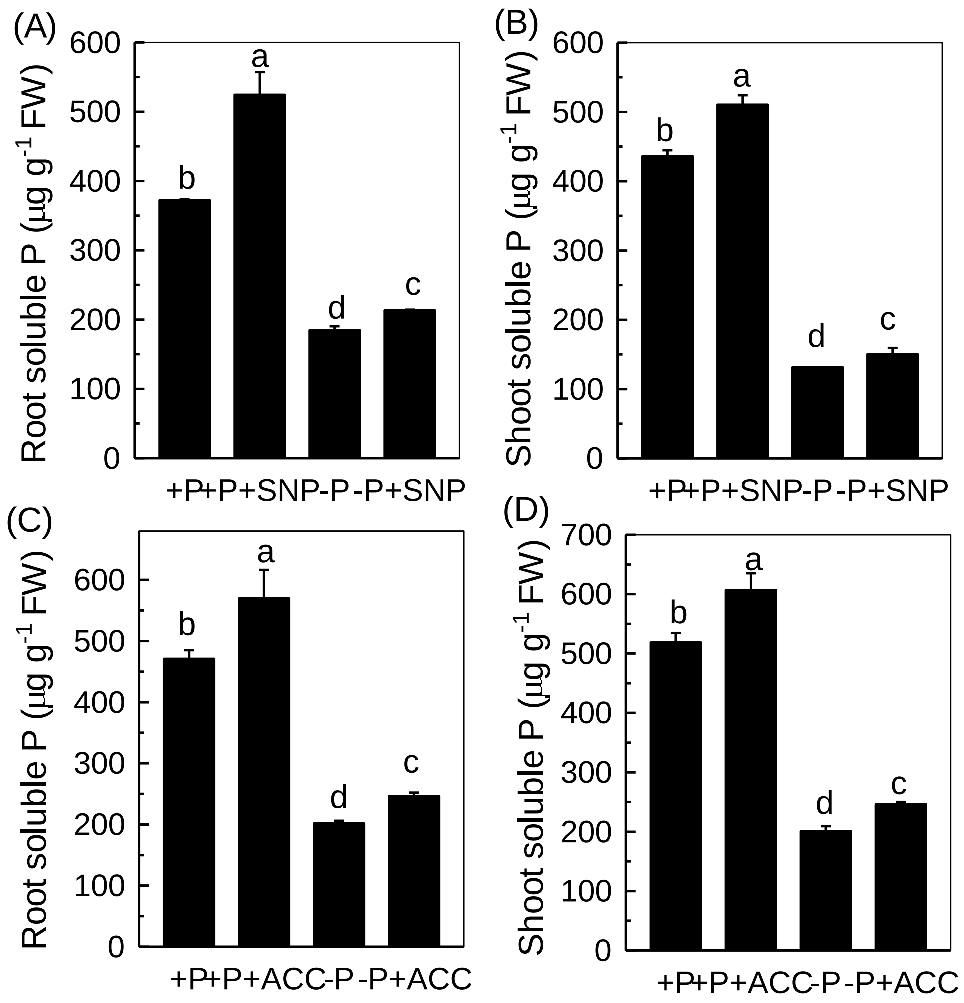

Supplemental Figure 2 Effect of SNP on root soluble P content (A), shoot soluble P content (B), and effect of ACC on root soluble P content (C) and shoot soluble P content (D) under +P or -P condition. Data are means ± SD (n = 4). Columns with different letters show significant differences at $P < 0.05$. FW: fresh weight.

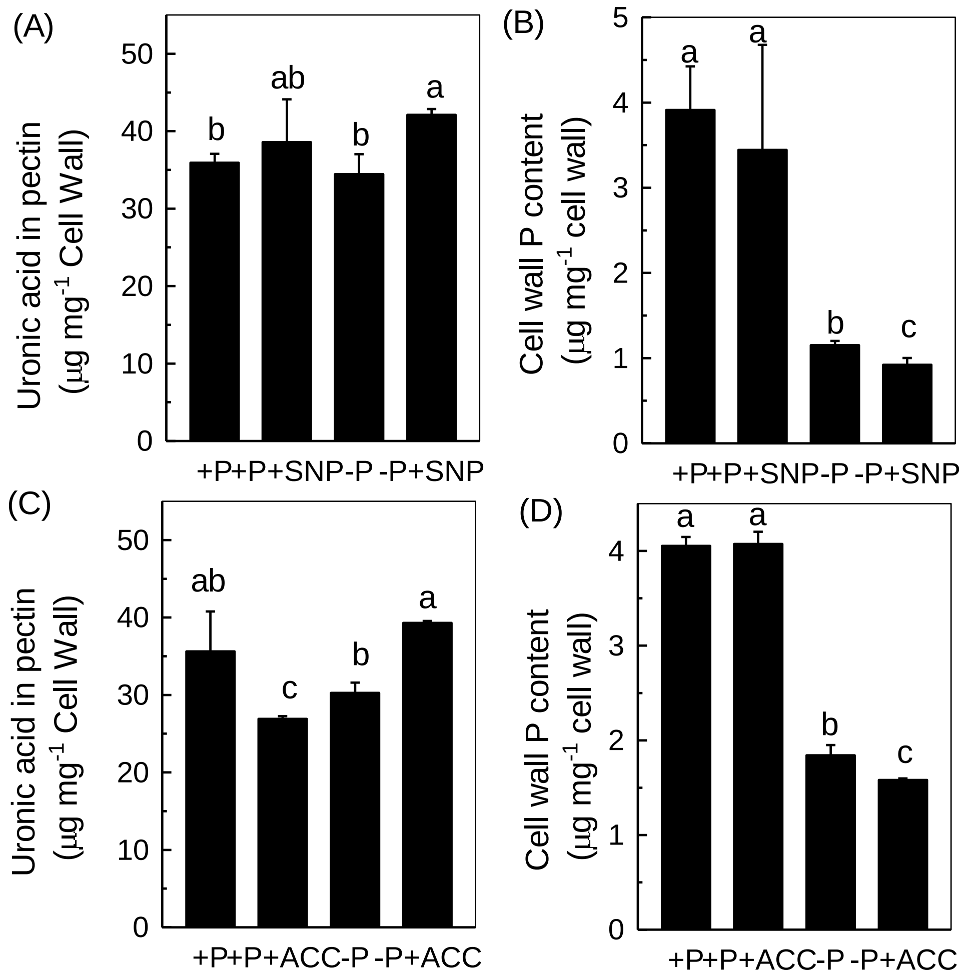

Supplemental Figure 3 Effect of SNP on cell wall pectin content (A), cell wall P content (B) and effect of ACC on cell wall pectin content (C), cell wall P content (D) in rice root under +P or -P condition. Data are means ± SD (n = 4). Columns with different letters show significant differences at $P < 0.05$.

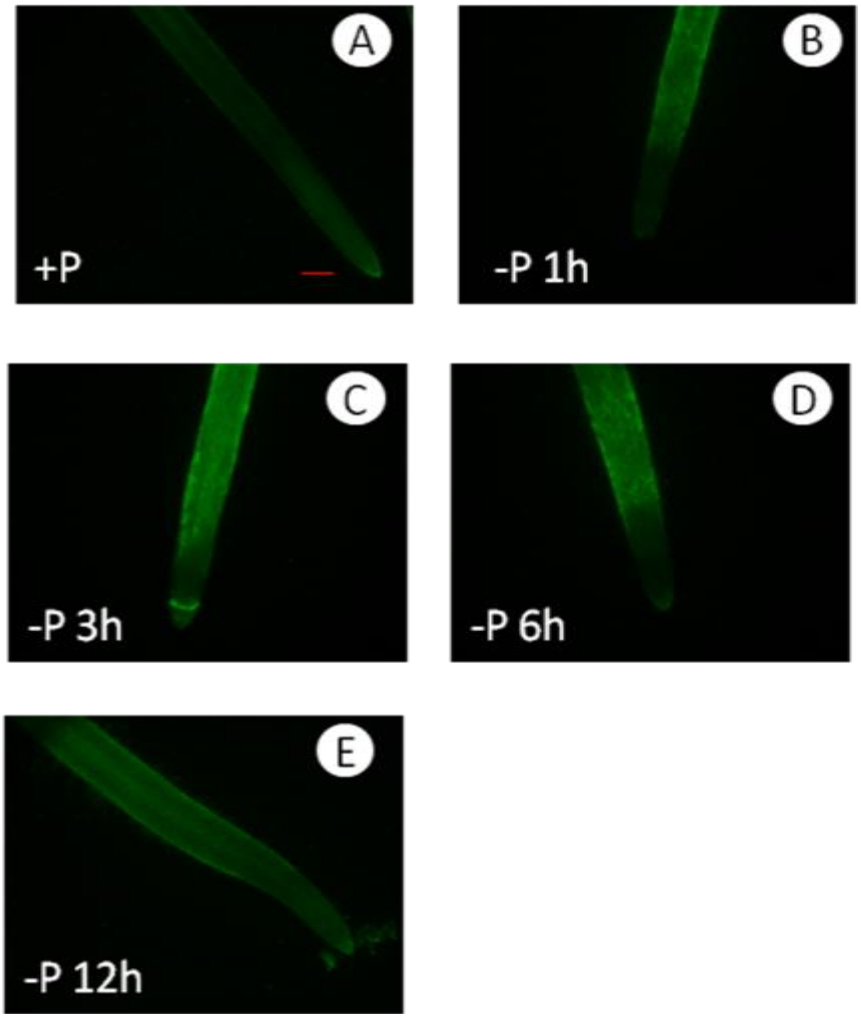

Supplemental Figure 4 Effect of P deficiency on NO production in rice root. Seedlings were first grown in the P sufficient condition as control (A), and then subjected to P deficient condition for 1 h (B), 3 h (C), 6 h (D) and 12 h (E). Root tips were collected for the NO fluorescence measurement. Data are means ± SD (n = 10). Scale bar = 1 mm.

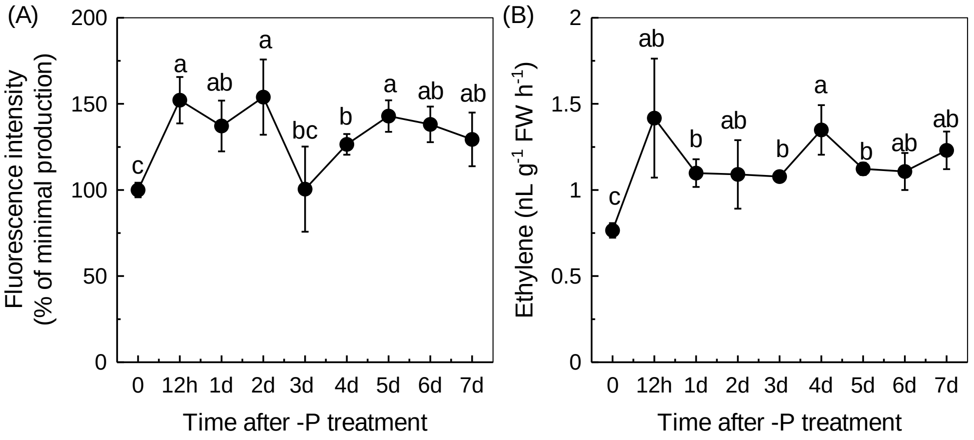

Supplemental Figure 5 Effect of P deficiency on NO production (A) and ethylene emission (B) in rice root. NO production is indicated by green fluorescence and expressed as relative fluorescence intensity (% of minimal production). Data are means ± SD (n = 10). Columns with different letters show significant differences at $P < 0.05$. FW: fresh weight.

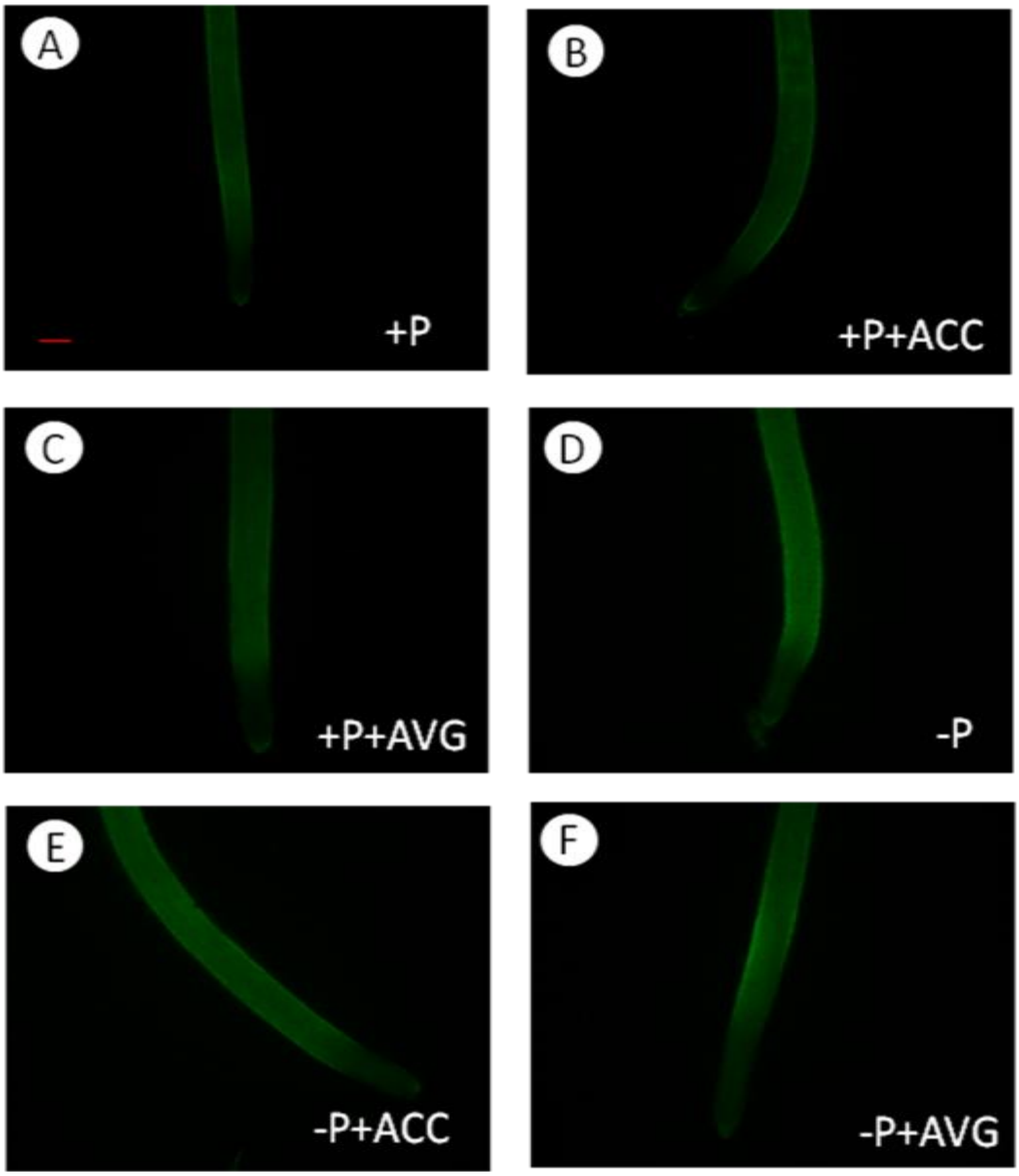

Supplemental Figure 6 Effect of different treatments on NO production in rice root under P sufficient (A, B and C) and P deficient condition (D, E and F). (B) and (D) seedlings were treated with 1 μM ACC, (C) and (F) seedlings were treated with 0.2 μM aminoethoxyvinylglycine (AVG). The root tips were collected for the NO fluorescence measurement. Data are means ± SD (n = 10). Scale bar = 1 mm.

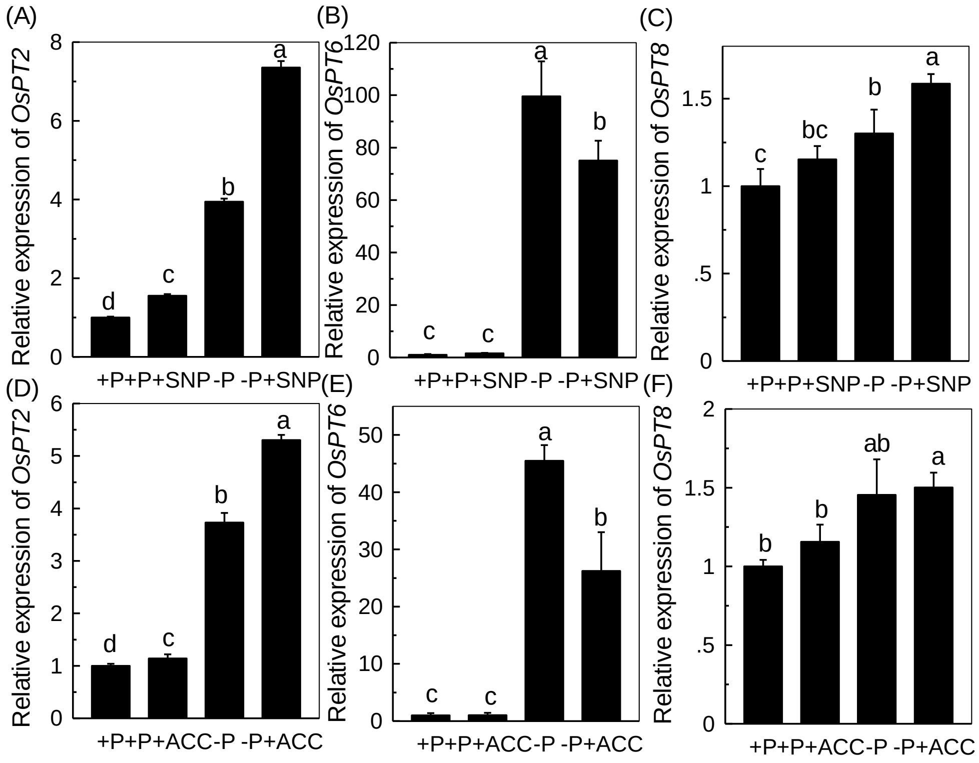

Supplemental Figure 7 Effect of SNP on the expression of *OsPT2* (A), *OsPT6* (B) and *OsPT8* (C), and effect ACC on the expression of *OsPT2* (D), *OsPT6* (E) and *OsPT8* (F) in rice roots under +P or -P condition. *OsHistone H3* was used as the reference gene. Data are means ± SD (n = 4). Columns with different letters show significant differences at $P < 0.05$.

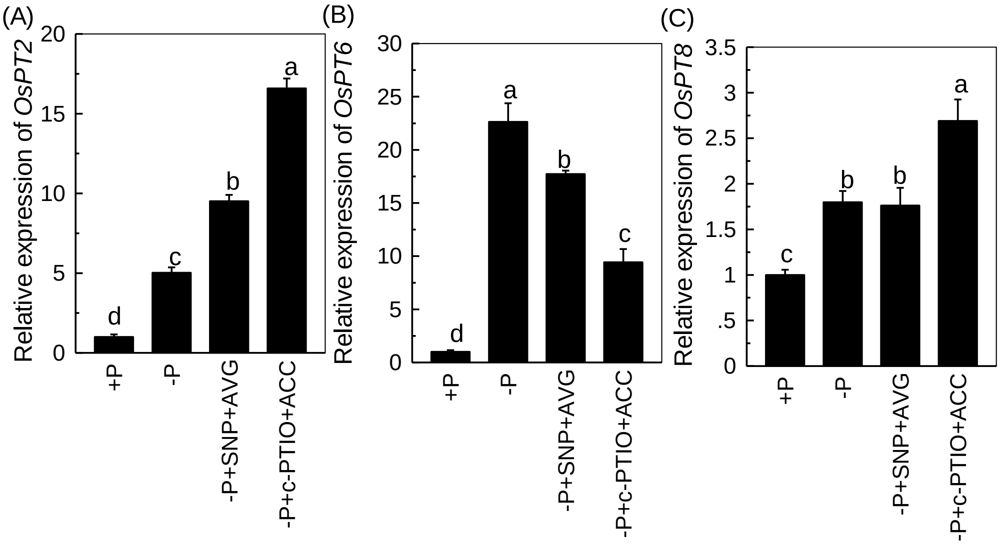

Supplemental Figure 8 Effect of different treatments on the expression of *OsPT2* (A), *OsPT6* (B) and *OsPT8* (C) in P-deficient rice. *OsHistone H3* was used as the reference gene. Data are means ± SD (n = 4). Columns with different letters show significant differences at $P < 0.05$.
